# StrainPanDA: Linked reconstruction of strain composition and gene content profiles via pangenome‐based decomposition of metagenomic data

**DOI:** 10.1002/imt2.41

**Published:** 2022-08-01

**Authors:** Han Hu, Yuxiang Tan, Chenhao Li, Junyu Chen, Yan Kou, Zhenjiang Zech Xu, Yang‐Yu Liu, Yan Tan, Lei Dai

**Affiliations:** ^1^ CAS Key Laboratory of Quantitative Engineering Biology, Shenzhen Institute of Synthetic Biology Shenzhen Institutes of Advanced Technology, Chinese Academy of Sciences Shenzhen China; ^2^ Bioinformatics Department Xbiome, Scientific Research Building, Tsinghua High‐Tech Park Shenzhen China; ^3^ Center for Computational and Integrative Biology Massachusetts General Hospital and Harvard Medical School, Richard B. Simches Research Center Boston Massachusetts USA; ^4^ Department of Food Science and Technology, State Key Laboratory of Food Science and Technology Nanchang University Nanchang China; ^5^ Channing Division of Network Medicine, Department of Medicine Brigham and Women's Hospital and Harvard Medical School Boston Massachusetts USA

**Keywords:** gene content profile, metagenomics, microbiome, pangenome, strain analysis

## Abstract

Microbial strains of variable functional capacities coexist in microbiomes. Current bioinformatics methods of strain analysis cannot provide the direct linkage between strain composition and their gene contents from metagenomic data. Here we present *Strain*‐level *Pan*genome *D*ecomposition *A*nalysis (StrainPanDA), a novel method that uses the pangenome coverage profile of multiple metagenomic samples to simultaneously reconstruct the composition and gene content variation of coexisting strains in microbial communities. We systematically validate the accuracy and robustness of StrainPanDA using synthetic data sets. To demonstrate the power of gene‐centric strain profiling, we then apply StrainPanDA to analyze the gut microbiome samples of infants, as well as patients treated with fecal microbiota transplantation. We show that the linked reconstruction of strain composition and gene content profiles is critical for understanding the relationship between microbial adaptation and strain‐specific functions (e.g., nutrient utilization and pathogenicity). Finally, StrainPanDA has minimal requirements for computing resources and can be scaled to process multiple species in a community in parallel. In short, StrainPanDA can be applied to metagenomic data sets to detect the association between molecular functions and microbial/host phenotypes to formulate testable hypotheses and gain novel biological insights at the strain or subspecies level.

## Highlights

- *Strain*‐level *Pan*genome *D*ecomposition *A*nalysis (StrainPanDA) uses the pangenome coverage profile of multiple metagenomic samples to simultaneously reconstruct the composition and gene content variation of coexisting strains in microbial communities.
- StrainPanDA allows accurate and robust inference of strain composition and gene content profiles on synthetic data sets.
- Linked reconstruction of strain composition and gene content profiles provided by StrainPanDA furthers our understanding of the relationship between microbial adaptation and strain‐specific functions (e.g., nutrient utilization and pathogenicity).

## Graphical abstract

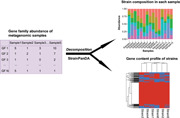

## INTRODUCTION

There is mounting evidence that multiple within‐species variants coexist in microbiomes [1, 2]. Coexisting microbial cells of the same species can have substantial variations in their gene contents (i.e., accessory genome), which is largely generated by horizontal gene transfer (HGT) [3, 4, 5]. The intraspecies variation in the accessory genome can lead to substantial phenotypic differences (e.g., nutrient utilization, pathogenicity, and antibiotic resistance) and plays an important role in microbial adaptation across environments [6, 7, 8, 9]. Moreover, many health outcomes linked to host‐associated microbiomes have been found to be consequences of the function of individual strains [8, 10, 11, 12, 13, 14].

Metagenomic sequencing has revolutionized microbiome studies by providing a culture‐independent approach to studying the composition and function of complex microbial communities. Commonly used tools for metagenomic analysis, known as metagenomics profilers, typically provide species‐level taxonomic composition [15, 16, 17, 18]. In parallel with the rapid increase of sequenced microbial isolates from culturomics studies [1, 9, 19, 20], high‐resolution analyses of metagenomic data have revealed notable within‐species variations [21, 22]. Methods that enable strain‐level analysis of metagenomes have been used for tracking strain transmission or dispersal [23, 24], studying the population genetics of microbial strains [25], and typing strains of specific interest [26, 27, 28, 29, 30, 31, 32].

The gene content profile of a microbial strain determines its biological function. To date, the majority of strain‐level analysis methods use single nucleotide variants (SNVs) to identify strain composition [25, 28, 29, 33, 34]. By assuming an association between SNV haplotypes and gene content profiles [4], SNV‐based methods can indirectly profile the within‐species gene content variation. However, for many species, it has been shown that SNV haplotypes cannot capture microbial genetic diversification resulting from HGT [4]. Alternatively, the current pangenome‐based method can infer the gene content of the dominant strain in a metagenomic sample [35] but fails to provide the abundance and gene contents of coexisting strains within the sample. Establishing the linkage of composition and gene contents of coexisting within‐species variants can provide crucial insights into microbial adaptation and microbiome–host interactions, but is inaccessible from currently available reference‐based bioinformatics tools.

**Figure 1:**
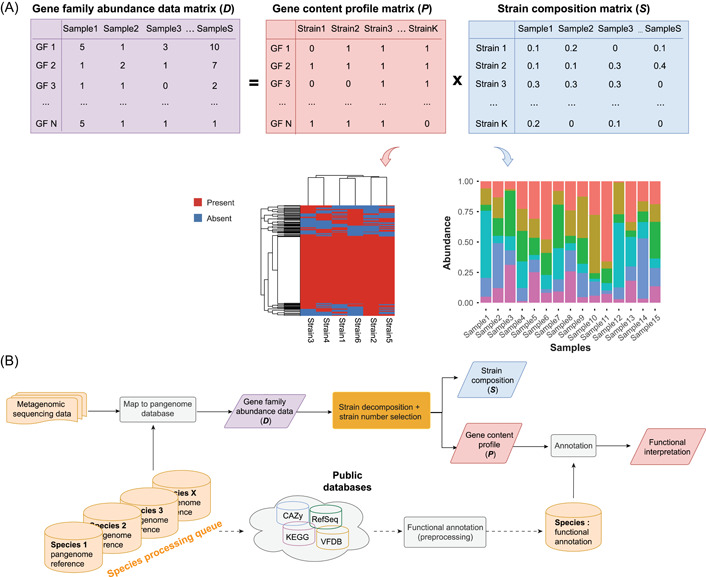
Illustration of the StrainPanDA workflow. (A) The gene family abundance data matrix ***D*** is decomposed into the product of two matrices ***P*** and ***S*** via nonnegative matrix factorization (Methods section). The gene content profile matrix ***P*** is a binary matrix that denotes the presence/absence of gene families in each strain. The strain composition matrix ***S*** represents the relative abundance of coexisting strains in each sample. In the illustrated example, the size of metagenomic samples *S* = 15 and the number of strains *K* = 6. (B) The workflow of StrainPanDA analysis is performed in a species‐by‐species manner, including mapping metagenomic reads to the pangenome database, strain decomposition, and functional annotation of gene family profiles. CAZy, Carbohydrate‐Active enZYmes; GF, gene family; KEGG, Kyoto Encyclopedia of Genes and Genomes; StrainPanDA, *Strain*‐level *Pan*genome *D*ecomposition *A*nalysis; VFDB, Virulence Factor Database.

To meet the increasing needs of strain‐level functional inference from metagenomics data, we developed a novel method known as *Strain*‐level *Pan*genome *D*ecomposition *A*nalysis (StrainPanDA) to simultaneously reconstruct the composition and gene contents of coexisting strains using the pangenome coverage profile from metagenomic data (Figure 1). We validated the performance of StrainPanDA with a comprehensive collection of synthetic data sets and showed that StrainPanDA was able to accurately infer strain composition and gene content profiles from metagenomic data. To demonstrate the practical use of StrainPanDA in human microbiome studies, we analyzed longitudinal gut microbiome samples of mother–infant pairs [36] and patients treated with fecal microbiota transplantation (FMT) [29, 37]. We found that StrainPanDA was able to identify the association between strain‐specific functions and microbial adaptation (or host phenotypes), leading to novel biological insights at the infraspecific level and testable hypotheses of molecular mechanisms.

## RESULTS

### Decomposition of the pangenome coverage profile to infer strain composition and gene content

The pangenome coverage profile of a microbial species from metagenomic data is composed of the gene contents of all coexisting strains. If there are multiple metagenomic samples with varying strain compositions, in principle it is possible to infer the composition of strains within the sample as well as the gene contents of each strain from the pangenome coverage profile [38]. Building on this intuition, the main algorithm of StrainPanDA aims to decompose the gene family abundance data matrix ***D*** into the product of two matrices, the gene content profile matrix ***P***, and the strain composition matrix ***S*** (Figure 1A, see Methods section for details). Here the pangenome coverage profile from metagenomic data is represented by matrix ***D***, where $D_{\mathrm{ij}}$ is the normalized count of gene family i in metagenomic sample j. The gene contents of coexisting strains are represented by binary matrix P, where the element $P_{\mathrm{ij}}$ indicates the presence/absence of gene family i in strain j. The composition of coexisting strains across samples is represented by S, where $S_{\mathrm{ij}}$ is the relative abundance of strain i in sample j $\left(S_{ij}\geq 0\;\text{and}\;\sum_{i}S_{ij}=1\right)$. In the implementation of StrainPanDA, the gene family abundance matrix ***D*** is decomposed by nonnegative matrix factorization (NMF) [39, 40, 41] to solve for matrices ***P*** and ***S***. This processing allows StrainPanDA to simultaneously delineate the composition and gene contents variation of coexisting strains.

The StrainPanDA software provides a fully automated workflow of strain analysis (Figure 1B), which imports raw sequencing data from multiple metagenomic samples, and performs reads mapping, strain decomposition, and downstream annotations (see Methods section for details). To assist the interpretation of gene content variation in strains, StrainPanDA incorporates functional annotation from several databases, including but not limited to Kyoto Encyclopedia of Genes and Genomes (KEGG) [42], Carbohydrate‐Active enZYmes (CAZy) [43], and Virulence Factor Database (VFDB) [44].

### StrainPanDA provides accurate predictions of strain composition and gene family profiles in synthetic data

**Figure 2:**
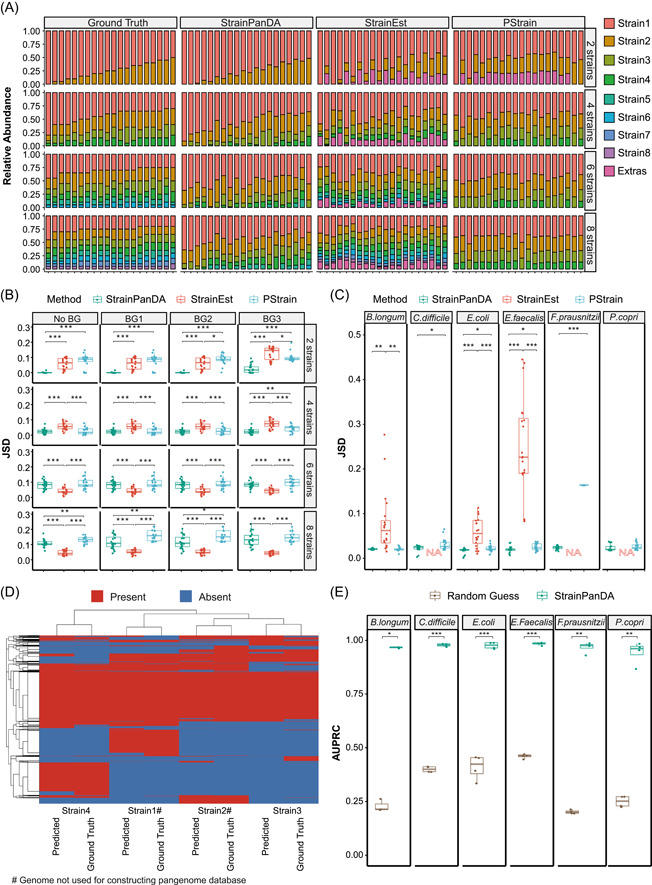
Validation of StrainPanDA using synthetic metagenomic data. (A) Comparison between the actual strain composition (Ground Truth) and the strain composition predicted by StrainPanDA and existing tools (StrainEst and PStrain) in synthetic mixtures of *Escherichia coli* strains (pWGS data set, 1× sequencing depth, see Methods section). The number of actual *E. coli* strains in the mixture (*n* = 2, 4, 6, and 8) is shown in rows. Each stacked bar is one simulated sample. Strains are displayed by the order of sorted relative abundance. If the number of predicted strains exceeds the number of actual strains, the extra strains are grouped into “Extras.” (B) Jensen–Shannon Divergence (JSD) between the actual and predicted strain composition. No BG, No Background; BG1/BG2/BG3, synthetic data of *E. coli* strains mixed with three different metagenomic data sets as background (WGSBG data set, 100‐fold background, see Methods section). Each dot represents one simulated sample (*n* = 20). (C) JSD between the actual and predicted strain composition is evaluated for different microbial species. Each dot represents one simulated sample (*n* = 24). Outputs not available are marked as “NA.” (D) The reference and predicted gene family profiles of *E. coli* strains (the synthetic data used are the same as panel A). Each row is one gene family, and each column is one strain. Hierarchical clustering is based on Euclidean distance. (E) The area under the Precision‐Recall Curve (AUPRC) for the gene family profiles of coexisting strains is evaluated for different microbial species. Each dot represents the AUPRC of one strain (*n* = 4 strains). Brown dots represent random guesses of gene family profiles (see Methods section). *p* values from paired *t* test: *p < 0.05, **p < 0.01, and ***p < 0.001. *Bifidobacterium longum*, *Clostridium difficile*, *Enterococcus faecalis*, *Faecalibacterium prausnitzii*, and *Prevotella copri*. pWGS, pure whole genome sequencing; StrainPanDA, Strain‐level Pangenome Decomposition Analysis; WGSBG, whole genome sequencing background.

We validated the performance of StrainPanDA using synthetic metagenomic data (Methods section). For synthetic mixtures of *Escherichia coli* strains (ranging from 2 to 8 strains, see Methods section), the strain composition predicted by StrainPanDA was overall close to the actual composition (Ground Truth; Figure 2A), and its performance was better at a lower number of coexisting strains (2 and 4 strains). For quantitative comparison, we calculated the Jensen–Shannon divergence (JSD) and Matthews Correlation Coefficient (MCC) between the predicted and actual strain composition of simulated samples. JSD and MCC have been widely used in the evaluation of strain analysis tools [28, 30, 33]. At a lower number of coexisting strains (2 and 4 strains), the predicted strain composition by StrainPanDA was better than the state‐of‐the‐art SNV‐based methods, including StrainEst [30] and PStrain [33] (the latter was modified based on ConStrains [28]). While the results of StrainEst tended to include false positives at a lower number of coexisting strains, its performance was better at 6 and 8 strains. Furthermore, we generated synthetic mixtures of *E. coli* strains with varying levels of sequencing errors (Supporting Information Figure S2), sequencing depths (Supporting Information Figure S3), and different background noises (mixed with different metagenomic data sets, Supporting Information Table S1 and Figure S4). In comparison to SNV‐based methods, the performance of StrainPanDA in predicting strain composition was robust.

To evaluate the performance of StrainPanDA in different bacterial species, we generated synthetic data for common human gut bacterial species (*Bifidobacterium longum*, *Clostridium difficile*, *Enterococcus faecalis*, *Faecalibacterium prausnitzii*, and *Prevotella copri*; Supporting Information Table S2, see Methods section). In comparison to other methods, StrainPanDA made the most accurate prediction of strain composition across all species when strain number was 4 (with JSD as 0.021 ± 0.006; Figure 2C).

While current SNV‐based methods can reconstruct the composition of coexisting strains from metagenomic samples, they could not directly provide the gene contents of the predicted strains. Here we show that StrainPanDA allows simultaneous reconstruction of strain composition in each metagenomic sample and the gene content variations among strains. In synthetic mixtures of *E. coli* strains, the predicted gene family profiles by StrainPanDA were close to the actual profiles (Figure 2D, Supporting Information Figure S5; precision = 0.91–0.96, recall = 0.87–0.96, for the four strains in a synthetic mixture). In particular, we note that StrainPanDA is able to infer the gene family profile of strains not included in the prebuilt reference genome database. The area under the Precision‐Recall Curve (AUPRC) was over 0.95 for all *E. coli* strains, indicating that StrainPanDA was able to reconstruct the gene contents of microbial strains with high sensitivity and precision (Supporting Information Figure S6).

We further evaluated the predicted gene family profiles of the human gut bacterial species included in the synthetic data. The AUPRC was on average above 0.9 and significantly better than random guesses (Figure 2E). The predicted gene family profiles were robust to sequencing errors, sequencing depths, and the background of real metagenomic data (Supporting Information Table S4). Moreover, to demonstrate the ability of StrainPanDA to identify strain‐specific genes, the pathogenic *E. coli* outbreak strain O104 [45] was introduced in a synthetic mixture with other *E. coli* strains (Supporting Information Table S5). All outbreak‐related gene families were successfully recovered by StrainPanDA (Supporting Information Figure S7). Finally, the performance of StrainPanDA and Pangenome‐based Phylogenomic Analysis (PanPhlAn)/PanPhlAn3 in inferring the gene content profiles were comparable (Supporting Information Figure S8). We note that PanPhlAn and PanPhlAn3 can only report the gene content profile of the “dominant strain” in a particular metagenomic sample (i.e., the strain with the highest relative abundance); in contrast, StrainPanDA can identify the gene content profiles of all coexisting strains.

Taken together, our benchmarking results demonstrate that StrainPanDA provides accurate predictions of compositional profiles and gene contents of coexisting strains from metagenomic samples. In the following sections, we will demonstrate the application of StrainPanDA in two longitudinal metagenomic data sets to elucidate the diversity of the gut microbiome at the subspecies level.

### Succession of *B. longum* subspecies in infant gut microbiome is associated with breastfeeding patterns and the selection of nutrient utilization

The direct inference of both the population structure and gene content variation at the strain level is crucial to understanding the ecology of microbial communities. Here we apply StrainPanDA to study the adaptation of coexisting bacterial subspecies in the infant gut microbiomes. We analyzed a previously published data set that includes gut metagenomic samples from ~100 mother–infant pairs (infants were sampled at three time points: newborn, 4 months, and 12 months) [36] (Supporting Information Table S6). At the species level, the authors found that the composition of the infant gut microbiome had distinctive features at each sampled time point, and the cessation of breastfeeding was clearly associated with the maturation of an infant gut microbiome into an adult‐like microbiome [36].

**Figure 3:**
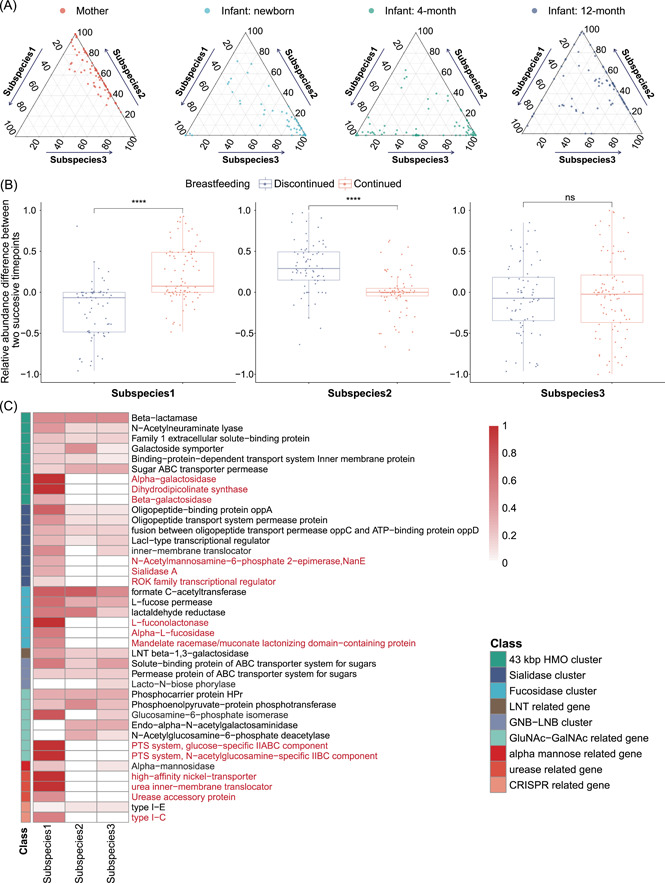
Succession of *Bifidobacterium longum* subspecies in infant gut microbiome can be attributed to the selection of nutrient utilization. (A) Ternary plots of the predicted composition of three subspecies of *B. longum* from mothers and infants of multiple time points (newborns, 4 months, and 12 months). Each dot represents one sample. (B) The shift in the relative abundance of *B. longum* subspecies between successive time points. According to the breastfeeding status at the subsequent time point, infants are divided into two groups (purple, discontinued breastfeeding, *N* = 86; red, continued breastfeeding, *N* = 71). ****p < 0.00005; ns, not significant; Student's *t* test. (C) Gene family profiles of predicted *B. longum* subspecies. Gene families related to the metabolism of host glycans, urease, and CRISPR (KEGG annotations) are selected for display. The color bar on the left indicates the class of gene clusters. Each row is a subclass of gene families and unique gene families of subspecies 1 are marked in red. The color scale in the heatmap indicates the normalized gene family coverage in the specific subclass (i.e., the fraction of detected gene families belonging to the subclass). HMO, human milk oligosaccharide; KEGG, Kyoto Encyclopedia of Genes and Genomes; ABC, ATP‐binding cassette transporters; ATP, adenosine triphosphate; CRISPR, clustered regularly interspaced short palindromic repeat; Gal, galactose; Gal‐NAc, *N*‐acetylgalactosamine; Glu‐NAc, *N*‐acetylglucosamine; GNB, galacto‐*N*‐biose; LNB, lacto‐*N*‐biose; LNT, lacto‐*N*‐tetraose; PTS, phophotransferase system; ROK, repressor, open reading frame, kinase.

We focused on the infraspecific analysis of *B. longum*, which is known to play an important role in the development of the infant gut microbiome [36, 46, 47, 48, 49, 50] and was found to be enriched in 4‐month infant samples in this study (Supporting Information Figure S9). Interestingly, we discovered a clear pattern of succession in the subspecies composition of *B. longum* over time, that is, a shift in the dominant subspecies (Figure 3A). Among the three *B. longum* subspecies predicted by StrainPanDA, *B. longum* subspecies 2 was dominant in the gut microbiomes of mothers. In the gut microbiomes of infants, *B. longum* subspecies 3 was most prevalent for newborns, while subspecies 1 transiently increased at the intermediate time point (at 4 months) and then was outcompeted by subspecies 2 (at 12 months). On the basis of the diet history provided in the original study, we further grouped infant samples into two different categories: “discontinued breastfeeding” and “continued breastfeeding” between successive time points (Methods section). It was evident that the relative abundance of *B. longum* subspecies 1 was enriched in infants that continued breastfeeding (Figure 3B). By contrast, once breastfeeding was discontinued, *B. longum* subspecies 1 was taken over by subspecies 2.

The association between *B. longum* subspecies composition and breastfeeding patterns suggests within‐species competition in nutrient utilization functions (Figure 3C). On the basis of functional annotations of KEGG [42] and CAZy [43], we found clear variations in nutrient utilization genes among the predicted *B. longum* subspecies. *B. longum* subspecies 1 had unique gene families (marked in red, Figure 3C) that are key enzymes related to human milk oligosaccharide (HMO), including galactosidase, *α*‐l‐fucosidase, sialidase, and their corresponding CAZy groups (GH33, GH29, and GH95; Supporting Information Figures S12 and S13 and Table S7). In addition, the urease‐related gene families were only found in the gene family profile of subspecies 1. Therefore, the unique functional potential of *B. longum* subspecies 1 in utilizing HMO and urea [51] from breast milk could confer a competitive advantage under breastfeeding, consistent with our observations of its transient dominance at 4 months in infant gut metagenomes.

Our finding is consistent with previous reports [52] on HMO utilization genes in some *B. longum* strains as well as observed changes in the frequency of *B. longum* subspecies *infantis* after weaning [4, 46, 47, 53, 54]. The functional profile of subspecies 1, in comparison to *B. longum* reference genomes, suggests that it may correspond to the *B. longum* subsp*. infantis* (Supporting Information Figure S14), which has been isolated from infants and known to be associated with breastfeeding [46, 55, 56].

Overall, we show that StrainPanDA is able to identify associations between strain‐specific functions (via reconstruction of gene contents) and adaptation (via reconstruction of strain composition), leading to novel biological insights and testable hypotheses about microbial ecology at the subspecies level.

### Analysis of post‐FMT gut metagenomes reveals individualized subspecies profiles and subspecies‐specific functions

**Figure 4:**
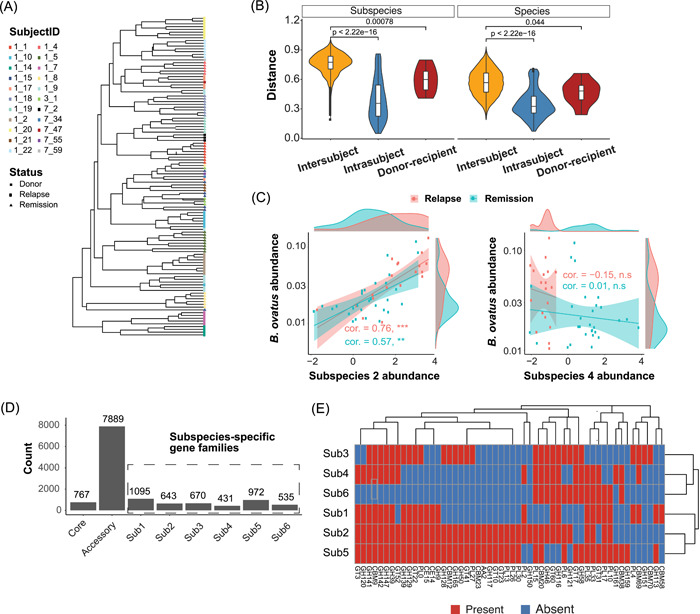
Analysis of post‐FMT gut metagenomes reveals individualized subspecies profiles and the association between subspecies‐specific functions and phenotypes. (A) Hierarchical clustering of predicted subspecies compositional profiles of common gut species (Supporting Information Table S8) reveals strong individual signatures. The subject IDs were collected from the original paper [37] and marked by different colors. (B) The dissimilarity (Bray–Curtis dissimilarity) in subspecies composition and species composition between samples. The pairs are classified into three groups for comparison: intersubject (samples from different individuals), intrasubject (samples from the same individuals), and donor–recipient (FMT donor vs. the post‐FMT sample of the recipient). (C) The relationship between the relative abundance of *Bacteroides ovatus* and its subspecies (normalized by centered log‐ratio transformation). Lines represent fitted linear regression (shaded areas: 95% confidence interval). The density plots on the side show the distribution of the corresponding variables. At the species level, *B. ovatus* is enriched in the relapse group. (D) The summary of pangenome information of predicted *B. ovatus* subspecies. (E) Gene family profiles of Carbohydrate‐Active enZYmes (CAZy) in predicted *B. ovatus* subspecies. CAZy genes shared by all the subspecies are not shown. FMT, fecal microbiota transplantation.

FMT introduces bacterial strains from healthy donors into recipients and has a profound impact on the structure and function of the recipient's gut microbiota [57, 58]. Here we apply StrainPanDA to analyze the metagenomic samples from FMT recipients in a clinical trial to treat Crohn's disease [37], including 17 patients (eight in the FMT group and nine in the sham group) and multiple samples (4–8 time points) for each patient. We analyzed the subspecies composition of commonly observed bacterial species in the human gut metagenome (Supporting Information Table S8). Hierarchical clustering of the predicted subspecies compositional profiles revealed strong individual signatures, which remained stable throughout 24 weeks (Figure 4A). The pairwise distance in subspecies composition profiles among samples of the same individual (sampled at multiple time points, that is, the “intrasubject” group) was significantly lower than the pairwise distance among samples of different individuals (i.e., the “intersubject” group; Figure 4B, *p* < 10^−15^), similar to the pattern at the species level. Furthermore, we found that the dissimilarity in subspecies composition between paired FMT donors and recipients (i.e., the “donor–recipient” group) was significantly lower than the “intersubject” group (*p* < 0.001), indicating the engraftment of donor strains and coexistence of donor and recipient strains [37]. We noted that the engraftment of donor gut bacteria was more obvious at the subspecies level (effect size = 1.4) than at the species level (effect size = 0.78; Figure 4B). Similarly, we applied StrainPanDA to analyze an independent FMT data set of metagenomic samples from patients with *C. difficile* infection [29]. We observed a clear pattern of subspecies engraftment in post‐FMT gut metagenomes, consistent with SNV‐based strain analysis in the original study [29] (Supporting Information Figure S15). Overall, we show that StrainPanDA is able to delineate the difference in subspecies composition among individuals and track the transmission of strains.

To elucidate the potential role of the gut microbiome in the maintenance of remission in Crohn's disease patients, we further investigated the strain‐level genetic signatures associated with post‐FMT clinical outcomes. The original study showed that the enrichment of Bacteroidetes species in patients relapsed after FMT [37]. We focused our analysis on *Bacteroides ovatus*, which was found to be enriched in relapsed individuals (false discovery rate [FDR]‐adjusted *p* = 0.2; Figure 4C–E). Among the predicted subspecies of *B. ovatus*, the relative abundance of subspecies 2 was positively correlated with the abundance of species‐level *B. ovatus* in gut metagenomes (Spearman correlation = 0.64, and FDR‐adjusted *p* < 10^−6^, Supporting Information Figure S16). We found substantial gene content variation among different *B. ovatus* subspecies (Figure 4D). Interestingly, we found that *B. ovatus* subspecies 2 had more CAZy family genes than others, indicating its functional potential to utilize diverse carbon sources and potential competitive advantages (Figure 4E). Thus, the strain‐specific metabolic functions of *B. ovatus* subspecies 2 may explain its dominance within the species (~20%) as well as its positive correlation with species abundance (Figure 4C and Supporting Information Figure S16). In addition, *B. ovatus* subspecies 2 carried several strain‐specific virulence factor genes (e.g., type IV secretion system and cholesterol‐dependent cytolysin), which may contribute to the positive association between *B. ovatus* and post‐FMT relapse (Supporting Information Figure S16). For example, cholesterol‐dependent cytolysin is a pore‐forming toxin that can disrupt the host plasma membrane [59], whose integrity has been linked to inflammatory bowel disease [60]. In addition, we noted that *B. ovatus* subspecies 4 was more abundant in the remission group (Figure 4C, FDR‐adjusted *p* < 10^−5^); whether this *B. ovatus* subspecies contributes to post‐FMT remission remains to be validated in future studies. Similarly, we performed StrainPanDA analysis for *Bacteroides vulgatus*, which was also enriched in relapsed individuals, and found clear functional variation among its subspecies (Supporting Information Figure S17).

In summary, we show that the linkage of strain composition and gene contents provided by StrainPanDA can greatly facilitate our understanding of microbial ecology beyond the species level. For microbes closely related to host health, this linkage helps formulate testable hypotheses on the association between molecular functions (e.g., pathogenicity) and clinical outcomes, which can be directly tested in experiments of isolated microbial strains.

## DISCUSSION

Here we report a novel method, StrainPanDA, to simultaneously profile the composition of coexisting strains and their corresponding gene content from metagenomics data. Our benchmarking results showed that StrainPanDA provided accurate and robust predictions from synthetic data. The predicted strain composition was better than or comparable to state‐of‐the‐art methods; meanwhile, the predicted gene content profile was close to the actual profile, even for strains not included in the prebuilt reference genome database. Furthermore, we applied StrainPanDA to metagenomic data sets to resolve within‐species variation of bacterial taxa of interest. For example, we found that the composition of *B. longum* subspecies in infant gut microbiomes was associated with dietary shifts, and the unique functional potential of certain *B. longum* subspecies in utilizing nutrients from breast milk might confer a competitive advantage. We demonstrated that the linkage of strain abundance and gene contents could lead to direct functional interpretations and testable hypotheses.

To study within‐species gene content variation, current SNV‐based methods implicitly assume the association between SNV haplotypes and gene content. However, many microbial genomes with high similarity in the core genome have less than 70% of genes in common [3], indicating that the indirect inference of gene content by SNV‐based methods may be insufficient. In contrast, StrainPanDA adopts the pangenome‐based approach to directly infer the gene content of multiple coexisting within‐species variants. Our current method relies on the pangenome constructed from a collection of genomes for a given microbial species, thus it does not account for gene content transfers between species. To account for interspecies gene transfer, one possible solution is to include a pool of “putative mobile elements” to expand the pangenome for each species. The prediction of StrainPanDA relies on the pangenome database, but it is not limited to the profiles of available reference genomes; thus, StrainPanDA can also be used to identify novel strains, as long as the relevant gene families are included in the pangenome. Although we focused on the comparison of StrainPanDA to other reference‐based methods, it is worth noting that complementary approaches based on metagenome‐assembled genomes (MAGs) can identify novel strains from metagenomic data. For example, DESMAN [61] can provide the predicted draft genome of each strain; other recently developed MAG‐based methods include mixtureS [62], STRONG [63], and so forth. In contrast to reference‐based methods, MAG‐based methods can identify novel species and genes, yet the quality of MAG will greatly affect the results. In addition, the MAG‐based methods require much higher sequencing depth than reference‐based methods, which prohibits their application to species with low abundance. In comparison, sequencing depth is not a limiting factor for StrainPanDA (Supporting Information Figures S3 and S4).

StrainPanDA is most suitable for the analysis of multiple metagenomic samples with shared within‐species variants, such as longitudinal studies. While the analysis in this study focused on the human gut microbiome, StrainPanDA is broadly applicable to microbiomes in different environments, as long as the pangenomes of the target species are available. The performance of StrainPanDA, including the accuracy of predicted strain composition and gene content profiles, improves with sample size (Supporting Information Figure S18) and sequencing depth (Supporting Information Figure S3). To apply StrainPanDA on a typical metagenomic data set, it would be desirable to have at least 10 samples and the relative abundance of the species of interest to be above 1%. Due to the nature of StrainPanDA's algorithm, it may be difficult to disentangle within‐species variants with genetic mosaic or highly similar gene content profiles (i.e., lacking strain‐unique features), thus StrainPanDA is most suitable for analysis at the level of subspecies [3]. Finally, in comparison to MAG‐based methods, StrainPanDA has minimal requirements for computing resources (Supporting Information Figure S19) and can be scaled to process multiple species in parallel.

## CONCLUSION

In summary, we show that StrainPanDA is able to provide accurate profiling of strain composition and gene content from metagenomic data. We envision that the application of StrainPanDA to the rapidly increasing metagenomic data sets, especially in the context of spatiotemporal characterization of microbiomes [64, 65, 66, 67], will help elucidate novel associations between molecular functions and microbial/host phenotypes as well as microbial ecology at the infraspecies level.

## METHODS

### Generation of pangenome database and mapping of metagenomic data

The pangenome database of bacterial species analyzed in this study was created following the steps recommended by PanPhlAn (version 1.2.8) [35]. For each bacterial species, genomes were downloaded from National Center for Biotechnology Information (NCBI). Average Nucleotide Identity (ANI) between genomes was calculated by mash (version 1.1) [68]. Representative strains (pairwise ANI ≤ 99%) were selected and used as reference genomes for pangenome construction. The annotated genes were extracted from the reference genomes and clustered into gene families at 95% identity by usearch (v7) [69] to create the pangenome database. Shotgun metagenomic data were mapped to the pangenome database by PanPhlAn [35] (version 1.2.8), which used Bowtie2 (version 2.4.1) [70] and SAMtools (version 0.1.19) [71] to map and count the reads, respectively. A gene family profile was generated by summing up the read counts of genes (normalized by reads per kilobase million [RPKM]) belonging to the same gene family. The gene family profiles of all metagenomic samples were grouped into a single gene family profile matrix. To account for potential noise in reads mapping, the gene family abundance was trimmed to 0 if the RPKM value was below the cutoff (10, by default). After trimming, gene families absent in all samples were removed from further analysis. In addition, samples were filtered out if the number of gene families detected was below 0.9 × *g*_min_ (*g*_min_ is the minimum number of gene families found in all reference genomes).

### StrainPanDA algorithm

The core algorithm of StrainPanDA decomposes the gene family abundance data matrix (D) of the microbial species of interest into the product of two matrices (Figure 1):

D=P ∙ S.

The gene family abundance data matrix D is an *N* × *S* nonnegative matrix, where $D_{\mathrm{ij}}$ is the normalized count of gene family i in metagenomic sample j. The gene content profile matrix P is an *N* × *K* binary matrix, where $P_{\mathrm{ij}}$ is 1 if the gene family i is present in strain j and 0 otherwise. The strain composition matrix S is a *K* × *S* matrix, where $S_{\mathrm{ij}}$ is the relative abundance of strain i in the sample $\left(S_{ij}\geq 0\;\mathrm{and}\;\sum_{i}S_{ij}=1\right)$. *N* is the number of gene families in the pangenome of the microbial species of interest, *S* is the number of metagenomic samples, and *K* is the number of strains (i.e., factorization rank).

To estimate ***P*** and ***S***, we approximate the solution ***P′*** and ***S′*** using NMF, considering the nonnegative constraints on both matrices (optimized using the “snmf/r” algorithm implemented in the R package “NMF” [40, 41], version 0.21.0). The addition of sparsity constraints (i.e., regularization terms in the objective function) ensures the uniqueness of factorization [41, 72]. The ***S′*** matrix is then scaled into relative abundances. We binarize the approximated ***P′*** matrix, following the assumption that the matrix elements corresponding to “present” gene families should have higher values than “absent” gene families, and the matrix elements should have a tight distribution due to the expectation that ***P*** is a binary matrix (see Supporting Information Figure S1B). Briefly, we find the peak of the probabilistic density curve (*p*_max_) for each strain *j*, where the number of matrix elements on the right of the peak ($P_{\mathrm{ij}}$ > *p*_max_) is equal to the expected number of gene families of the species of interest (i.e., averaged over all reference genomes in the pangenome database). We then cut the density curve at *θ* between the selected peak and 0 (*θ* = 0.5 × *p*_max_, by default), where the gene families with a weight greater than *θ* are considered as present. The confidence score *C*_*ij*_ for gene family *i* in sample *j* was assigned to every gene family:

$$C_{\mathrm{ij}}=\left\{\begin{array}{ll} 1, & p_{ij}^{\prime }\geq \theta ,\\ \frac{\theta -p_{ij}^{\prime }}{\theta }, & p_{ij}^{\prime }<\theta \end{array}\right..$$

### The confidence scores were used to rank gene presence predictions for generating the Precision‐Recall curves in the benchmarking experiments

To select the proper number of strains (i.e., the rank of NMF), we parsimoniously select the least number of strains from a range of 1–12 (by default) satisfying the following criteria: (1) The mean relative abundance across all the samples of any strain should be greater than *τ*_2_ (*τ*_2_ = 0.1 by default), (2) the number of gene families of all strains should be greater than *τ*_3 _× *g*_min_ (*τ*_3_ = 0.5 by default), *g*_min_ is the minimum number of gene families found in all reference genomes, and (3) the gene family profiles between a pair of strains should have Jaccard distance larger than *τ*_1_ (*τ*_1_ = 0.1 by default). The program also provides an option to accept a user‐specified number of strains set. In this study, we did not set the number of strains a priori in benchmarking and applications of StrainPanDA.

### Benchmarking StrainPanDA with synthetic data

#### Synthetic data of *E. coli* strains

We generated four types of simulated sequencing reads: (1) Error‐Free (ErrFree): pick random fragments from the reference genome of *E. coli* by read simulator ART [73] (version 2016.06.05; parameter: ‐ef ‐ss HS25 ‐l 150 ‐m 270 ‐s 27); (2) ART with sequencing errors (ARTErr): use ART to add sequencing errors on top of ErrFree reads (parameter: ‐ss HS25 ‐l 150 ‐m 270 ‐s 27); (3) pure whole genome sequencing (pWGS): randomly draw reads from the WGS data of selected strains by seq‐tk (https://github.com/lh3/seqtk, version 1.3; default parameter); and (4) pWGS data mixed with a real background metagenomic data set (whole genome sequencing background [WGSBG]): Three different metagenomic data sets (see Supporting Information Table S4; BG1, IBD; BG2, FMT; BG3, MI; as shown in Figure 2) were used to mix with the pWGS data of *E. coli* at different ratios (1‐, 5‐, 10‐, 25‐, and 100‐fold). Metagenomic samples were analyzed by Kraken2 (version 2.1.1; database: miniKraken2_v2_8GB_201904) to ensure a minimal abundance of *E. coli*. Strains of *E. coli* with pairwise genome‐wide ANI between 95%–99% were selected to represent different subspecies (Supporting Information Table S3). In each synthetic data set of mixed strains, 20 combinations of strain composition were generated by Dirichlet distribution (Supporting Information Table S2). All synthetic data sets were generated by the SimStr pipeline (https://github.com/xbiome/StrainPanDA/tree/main/SimStr). For each strain, its genome size was considered 1× sequencing depth and used to calculate the number of reads to generate. The minimum relative abundance (i.e., frequency) of a strain was set as 5% and as one unit. For example, for *E. coli* synthetic data of 1× sequencing depth that we refer to in this study, the data size of the strain with 5% frequency was ~4.5 megabases (MB), while the total depth of each sample in this data set was always 20×, and ~90 MB in size (1× sequencing depth as a unit and 20 units in total). To evaluate the effect of sample size, 400 synthetic mixtures of four strains were generated by Dirichlet distribution. The synthetic data were separated into 10 runs (40 samples each) and further downsampled to 20, 15, 10, and 5 samples in each run.

#### Synthetic data of gut bacterial species

Synthetic data of sixspecies, including *B. longum*, *C. difficile*, *E. coli*, *E. faecalis*, *F. prausnitzii*, and *P. copri*, were generated separately (Sync6, pWGS at 5× sequencing depth; Supporting Information Table S2). For each species, the relative abundances of four strains (5%, 10%, 25%, and 60%) were permutated to generate 24 samples in total (Supporting Information Table S2). All six species were in the prebuilt databases of StrainPanDA and PStrain. Only *B. longum*, *E. coli*, and *E. faecalis* were in the prebuilt database of StrainEst, so the other species were excluded in the comparison to StrainEst (Figure 2C).

### Evaluation of predicted strain composition

StrainEst (v1.2.4 through docker) and PStrain (downloaded from GitHub on May 23, 2021) were run with their prebuilt database and default parameters (StrainEst, ftp://ftp.fmach.it/metagenomics/strainest/ref/; PStrain, https://github.com/wshuai294/PStrain). The strain compositional profiles predicted by different methods were evaluated and compared by SimStr. For the predicted strain composition shown in Figure 2A (stacked bar plots), strains with relative abundance below 0.01 were filtered and the remaining strains were sorted by their relative abundance (rescaled to 1) in decreasing order. After sorting, the predicted strains in the lower tail exceeding the number of simulated strains were grouped into “Extras.”

Two commonly used metrics were used to evaluate the performance of predicted strain composition of different methods:

1. *JSD* [74]: JSD between the predicted strain composition and actual strain composition is calculated by the distance function in phyloseq [75] (R package) on the sorted relative abundance (in decreasing order). If the number of predicted strains is different from the actual number of strains, zeros were appended to the vector with a lower dimension. The JSD is symmetric and is in the interval of [0, 1]. It reflects the dissimilarity in compositional profiles of strains, that is, JSD = 0 represents an exact prediction.
2. *MCC* [76]:

$$\text{MCC}\;=\frac{\text{TP}\times \text{TN}-\text{FP}\times \text{FN}}{\sqrt{\left(\text{TP}+\text{FP})(\text{TP}+\text{FN})(\text{TN}+\text{FP})(\text{TN}+\text{FN}\right)}},$$

where TP is the number of true positives, TN is the number of true negatives, FP is the number of false positives, and FN is the number of false negatives. The MCC ranges from −1 to 1, where 1 represents an exact prediction, 0 represents a random prediction, and −1 represents total disagreement.

Owing to the lack of strain annotations from PStrain, we only computed MCC for strain composition predicted by StrainPanDA and StrainEst. For StrainEst, the predicted strains were directly annotated by reference genomes. For StrainPanDA, based on the predicted gene family profile, the Jaccard distance (JD(A,B)=1−|A∩B|÷|A∪B|) between the predicted strain and all reference genomes was calculated. The reference genome with the smallest Jaccard distance to the predicted strain was used for annotation. If a strain in the synthetic mixture is included in the prebuilt database of reference genomes, the ID of the annotated reference genome is directly compared with the actual strain to determine if the predicted strain is a true positive. If a strain in the synthetic mixture is not included in the prebuilt database of reference genomes, we used the phylogenetic tree to decide whether a predicted strain is a true positive (Supporting Information Table S4). Briefly, we generated a phylogenetic tree by parsnp [77] (version 1.5.1, default parameter) including genomes of the strains used in synthetic mixtures and all the reference genomes. If the annotated reference genome of a predicted strain is within the cutoff of phylogenetic distance (cutoff = 0.05 for *E. coli*, corresponding to ANI ~ 99%) from an actual strain, it is considered a true positive.

### Evaluation of predicted gene family profiles

For each microbial strain evaluated in benchmarking data sets, ErrFree reads at 5× sequencing depth were generated by ART simulator [73] (Sync‐Single data set) based on its reference genome downloaded from NCBI. The Sync‐Single data set contained three replicates for each strain and was used to generate the actual gene family profile of each strain by PanPhlAn and PanPhlAn3‐v3.1 (default parameters for sensitive mode). The gene families found in two or more replicates were considered “present.” The actual gene family profile (reference) of each strain was compared with the predicted gene family profile (Figure 2D). The Jaccard distances between microbial strains' predicted gene family profiles and their reference profiles (or the gene family profile of a randomly sampled reference genome) were computed (Supporting Information Figure S5). The Precision‐Recall curve of gene family profiles for each strain was generated by R package PRROC [78] using the confidence scores to rank the gene families predicted (Supporting Information Figure S6). For random guesses, 1000 random gene family profiles were generated by sampling *N* gene families from the pangenome as “present,” where *N* is the average number of gene families present in reference genomes. To demonstrate the ability of StrainPanDA to identify strain‐specific genes, the pathogenic *E. coli* strain *O*104 (GCF_002983645) was introduced in a synthetic data set of four strains (Sync *O*104 data set, pWGS, 5× sequencing depth). The outbreak‐related genes curated from Scholz et al. [35] were used to evaluate StrainPanDA's gene content prediction (Supporting Information Table S5). We also compared the predicted gene family profiles from StrainPanDA to the prediction from PanPhlAn and PanPhlAn3 (Supporting Information Figure S8).

### Runtime evaluation

The runtime of StrainPanDA was measured with the time command in Linux. All these tests were run on a workstation of Intel(R) Xeon(R) Gold 6238 CPU @ 2.10 GHz and 16 GB memory. The runtime (seconds) as a function of sample size was estimated by running StrainPanDA with the downsampled synthetic mixture of four *E. coli* strains (See *Synthetic data of E. coli strains* in Methods section). The runtime (seconds) as a function of strain number was calculated by running StrainPanDA with the pWGS and 25‐fold WGSBG data set.

### Applications of StrainPanDA in metagenomic data

#### Case study: Mother–infant gut metagenomes

All available samples of ERP005989 were downloaded from EBI (eight samples failed, Supporting Information Table S6). On the basis of the diet history, infants without diet history were filtered. The rest of the 84 infants were split into three different groups (B_F_F, discontinued breastfeeding at 4 months; B_B_F, discontinued breastfeeding at 12 months; B_B_B, continued breastfeeding; the F_B_M sample was excluded; Supporting Information Table S6). Samples without enough coverage on *B. longum* gene families were filtered by StrainPanDA at the preprocessing step and excluded from the downstream analysis. In the “continued breastfeeding” group, infants that kept breastfeeding between successive time points (i.e., between newborn and 4 months, or between 4 and 12 months) were included. In the “discontinued breastfeeding” group, infants who stopped breastfeeding by 4 or 12 months were included. For functional interpretation of the subspecies, we grouped the gene families annotated to the same KEGG (downloaded September 1, 2021) ortholog or CAZy (downloaded July 31, 2019) family. To further analyze the key functions related to breastfeeding, we curated a set of KEGG orthologs from related references [51, 79]. All the KEGG orthologs were further grouped into subclasses and classes based on the literature [51, 79] (Supporting Information Table S7). The gene family coverage was calculated as the fraction of detected genes belonging to the subclass (Figure 3C).

#### Case study: FMT donor–recipient metagenomes

Raw sequencing reads were downloaded from ENA (Accession: PRJNA625520 for the study on Crohn's disease [37], PRJEB23524 for the study on *C. difficile* infection study [29]). For Crohn's disease data set, species relative abundances were estimated using Kraken2 [18] (version 2.0.8‐beta) with the miniKraken database (v2_8GB_201904_UPDATE). To identify species associated with remission or relapse, the Wilcoxon rank‐sum test was conducted to select differentially abundant species using the mean relative abundances across different time points for each subject (samples collected before FMT or after relapse were discarded). The relative abundances of subspecies were predicted by StrainPanDA and normalized by the centered‐log‐ratio transformation for calculating Spearman correlation with the species abundances. For functional annotation of the subspecies, virulence factors and CAZy annotations were taken from the species‐specific databases constructed as described above.

### Functional annotation of gene families

To annotate the gene families by KEGG, gene family representative sequences were mapped against KEGG orthologs (release 2020‐07‐20) using usearch [69] (v11.0.667; “‐ublast”). Alignments with identity >50% and query coverage >50% are kept. To annotate the gene families by CAZy, SeqKit [80] (0.15.0) was used to translate the gene family centroids into six open reading frames. The translated amino acid was used as the input of run_dbcan [81] (2.0.11), which used DIAMOND [82] (2.0.8), HMMer [83] (3.3.2), and Hotpep [84] (2.0.8) with default parameters to predict the CAZy annotation. The CAZy annotations were selected only if it was predicted by at least two programs. If a gene family was assigned by more than one CAZy annotation, only the first annotation was used. To annotate virulence factors, gene family centroids were mapped against the VFDB (April 9, 2021) by DIAMOND [82] (2.0.8; blastp, query coverage >50% and identity >50%).

## AUTHOR CONTRIBUTIONS

Han Hu, Yan Tan, and Lei Dai conceived and supervised the study. Han Hu, Yuxiang Tan, and Chenhao Li developed the algorithm and performed the analysis on simulated and real metagenomic samples. Lei Dai, Han Hu, Yuxiang Tan, and Chenhao Li wrote the manuscript with inputs from Zhenjiang Zech Xu, Yang‐Yu Liu, Yan Kou, and Yan Tan.

## CONFLICTS OF INTEREST

The results described in this manuscript support pending patent application CN202011146154.3A. Yan Tan is cofounder and shareholder with a personal financial interest in Xbiome. Han Hu and Yan Kou are employees of Xbiome. Lei Dai received research grant support from Xbiome and serves as an unpaid consultant to the company. The remaining  author/authors declares/declare no conflicts of interest.

## Supporting information

Supporting information.

Supporting information.

## Data Availability

The source code of StrainPanDA is available on GitHub (https://github.com/xbiome/StrainPanDA) and Zenodo (https://doi.org/10.5281/zenodo.6668661). The experiment source code used in the manuscript is available from https://github.com/xbiome/StrainPanDA-data/tree/main/example#readme. Supplementary materials (figures, tables, scripts, graphical abstract, slides, videos, Chinese translated version, and update materials) may be found in the online DOI or iMeta Science http://www.imeta.science/.
