# Supplementary material for: StrainPanDA: Linked reconstruction of strain composition and gene content profiles via pangenome‐based decomposition of metagenomic data
Source: Imeta. 2022 Aug 1;1(3):e41. doi: 10.1002/imt2.41 (PMC10989911; doi:10.1002/imt2.41)
Supplement: Supplementary file 1 — Supporting information. [file IMT2-1-e41-s002.docx]

Supplementary Figures

Figure S1. Details of StrainPanDA algorithm. (A) Illustration of the determination of strain number (*i.e.*, factorization rank) in non-negative matrix factorization. The gene family abundance matrix *D* was decomposed into the product of a gene content profile matrix *P* and a strain composition matrix *S* at different number of strains (K=1 to *K_m_*, *K_m_*=15 by default). The rank selection step chooses a proper K (see Methods for details). (B) Illustration of the determination of gene family presence (*P* matrix). A threshold (vertical line) is used to determine the presence/absence of the gene families and confidence scores are calculated as described in Methods.


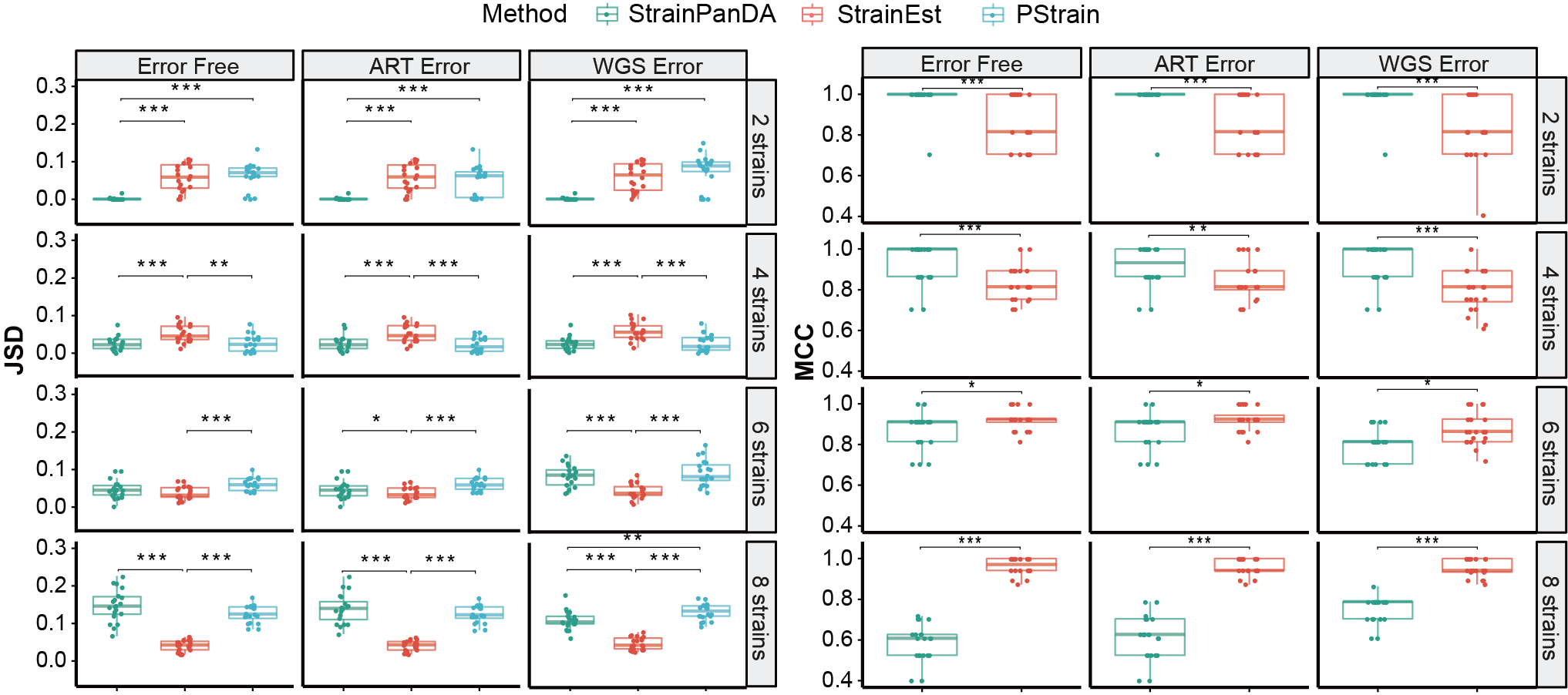


Figure S2. Comparison of predicted *E. coli* strain compositions among StrainPanDA, StrainEst, and PStrain using error free data (Error Free), data with sequencing error simulated using ART software (ART Error), and real sequencing data for the corresponding ground truth isolate (WGS Error, or “pWGS”) at 1× sequencing depth. JSD, Jensen-Shannon divergence. MCC, Matthews Correlation Coefficient. P values from paired *t*-test: *P < 0.05, **P < 0.01, ***P < 0.001.


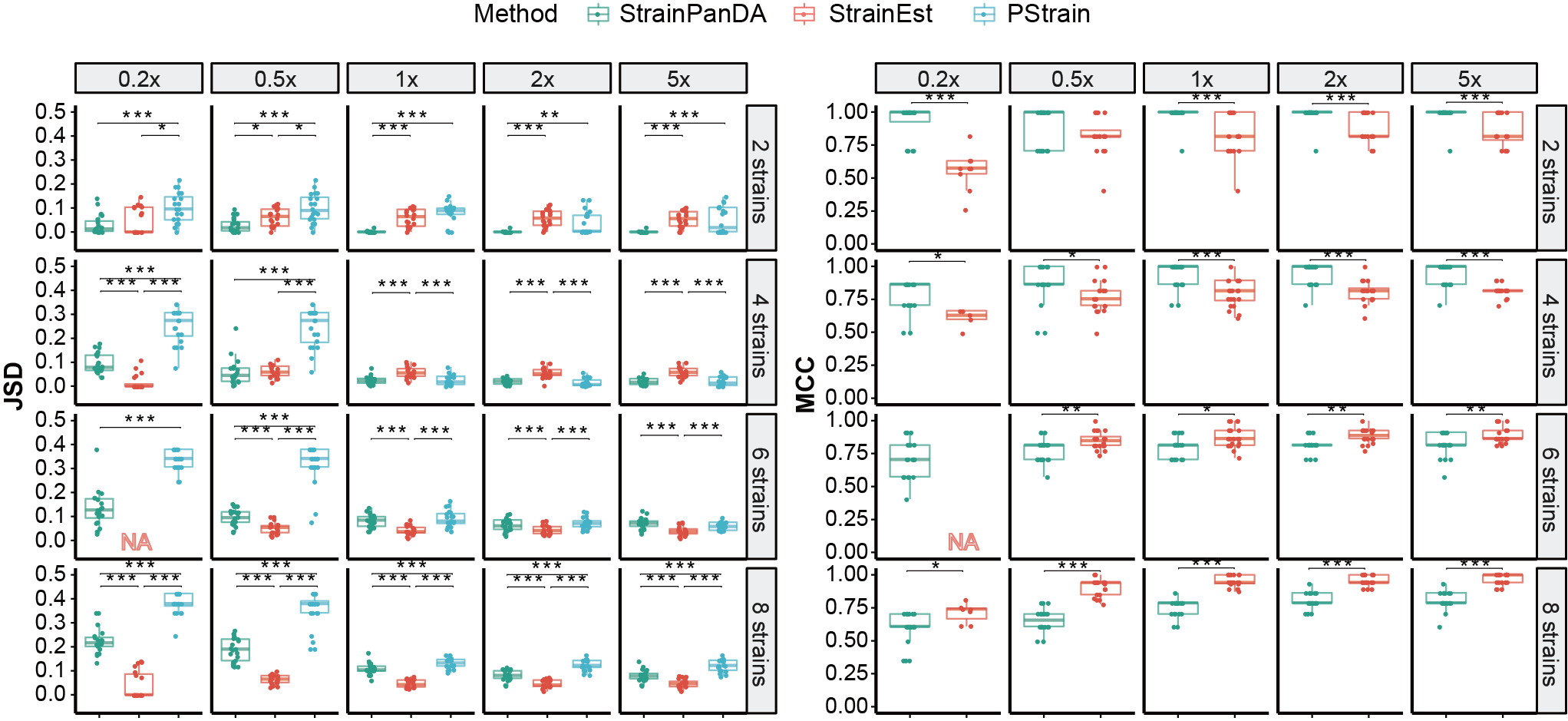


Figure S3. Comparison of predicted *E. coli* strain compositions among StrainPanDA, StrainEst, and PStrain using pWGS datasets of 0.2×, 0.5×, 1×, 2× and 5× sequencing depth. Output not available is marked as “NA”. MCC was not available for PStrain due to its lack of strain annotation. JSD, Jensen-Shannon divergence. MCC, Matthews Correlation Coefficient. P values from paired *t*-test: *P < 0.05, **P < 0.01, ***P < 0.001.


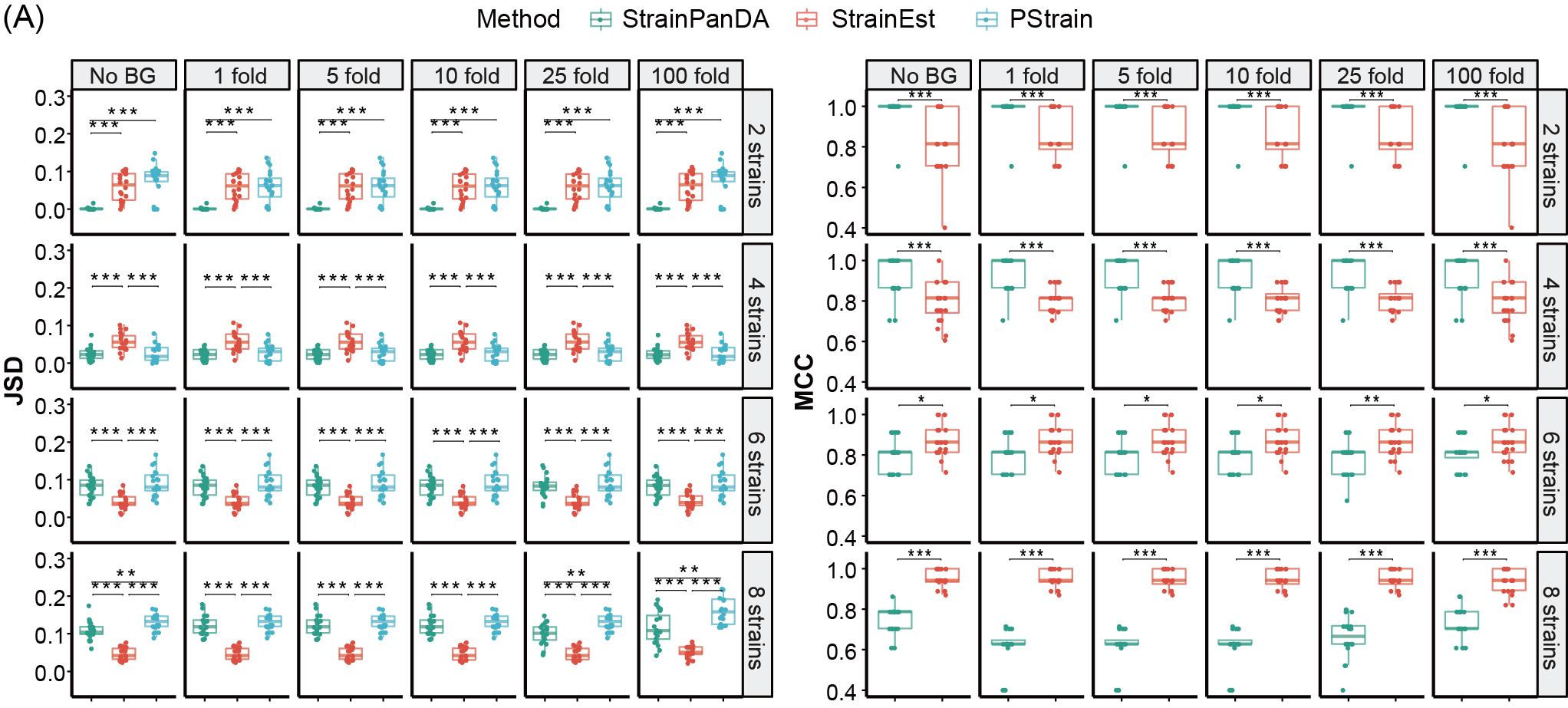

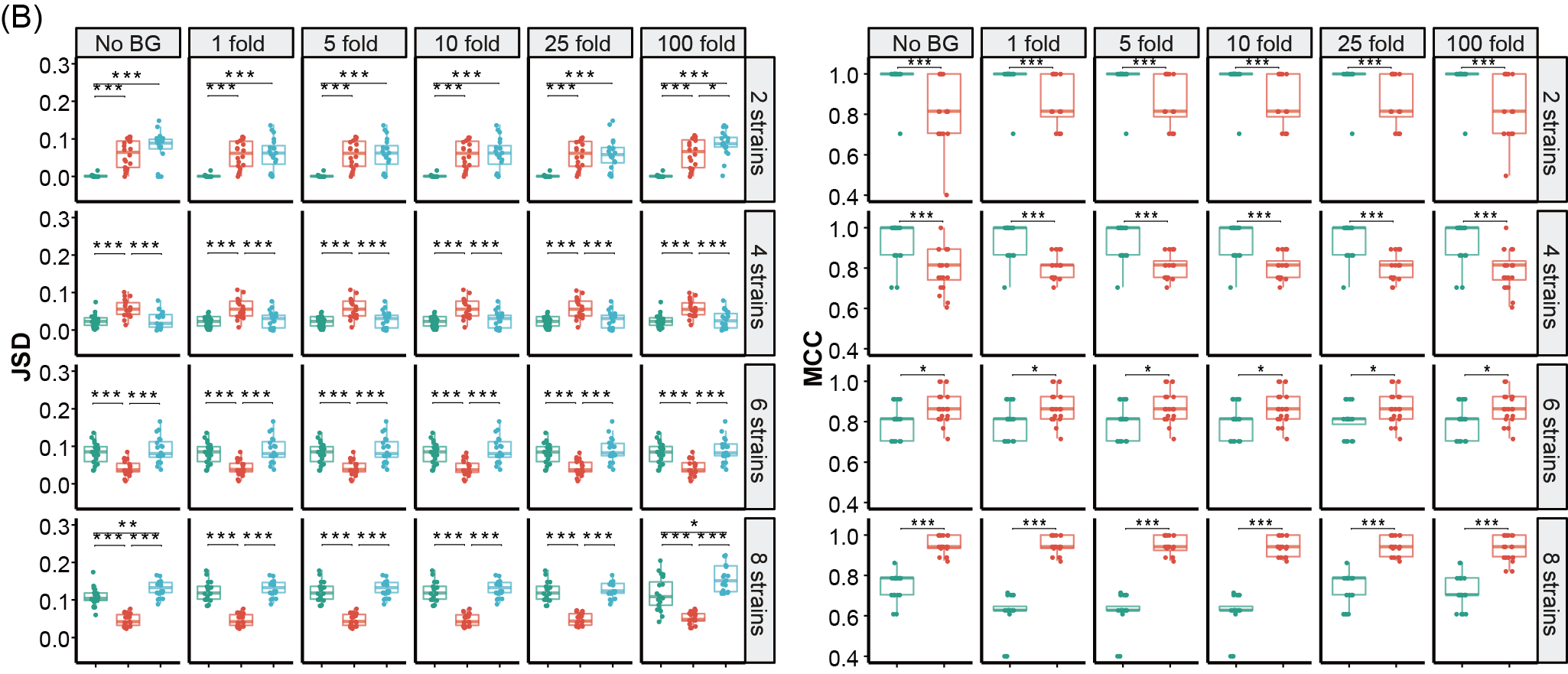

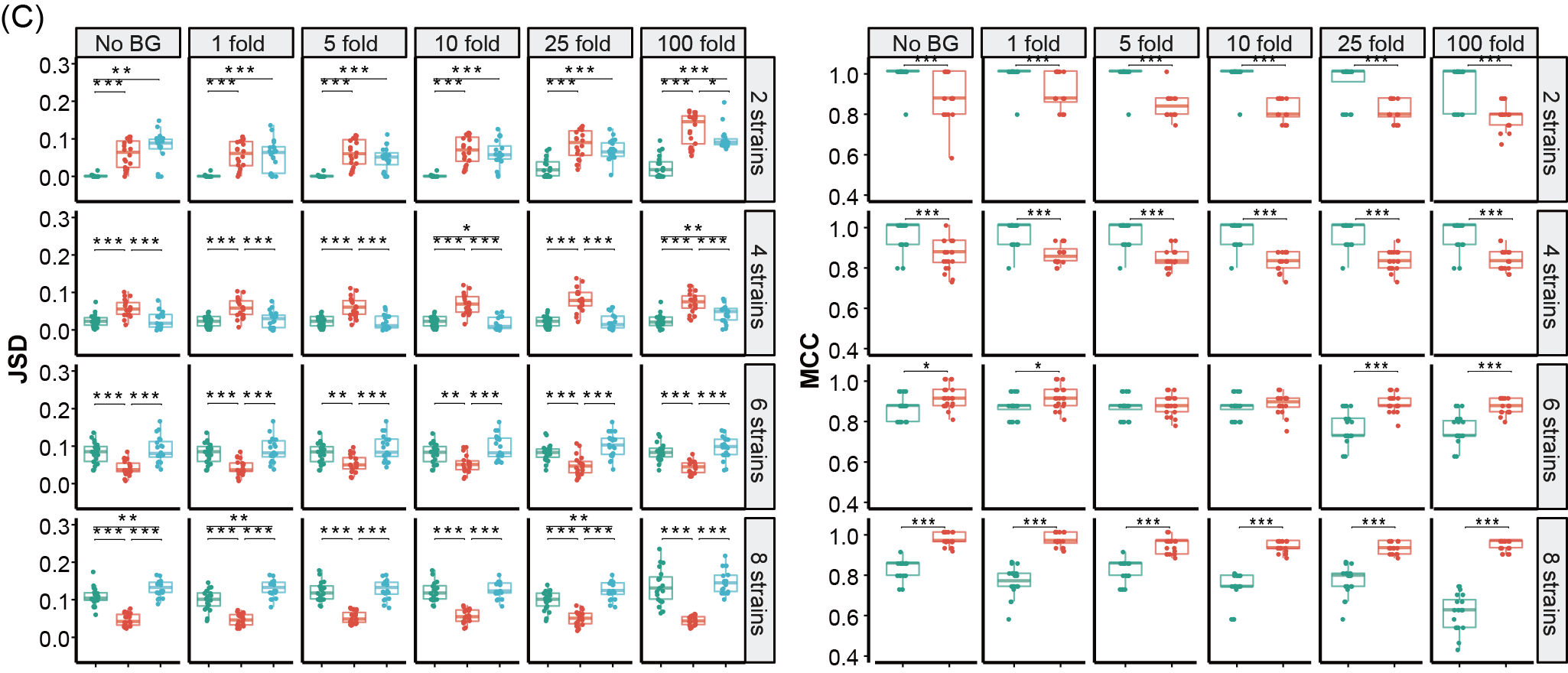


Figure S4. Comparison of predicted *E. coli* strain compositions among StrainPanDA, StrainEst, and PStrain on datasets of 1× sequencing depth and spike-in *E. coli* WGS reads with 1-fold, 5-fold, 10-fold, 25-fold and 100-fold background reads from public datasets. The datasets include the inflammatory bowel disease (panel A) (https://ibdmdb.org/), fecal microbiota transplantation (panel B) (Smillie *et al.* 2018) and mother-infant (panel C) (Bäckhed *et al.* 2015) datasets. MCC was not available for PStrain due to its lack of strain annotations. JSD, Jensen-Shannon divergence. MCC, Matthews Correlation Coefficient. P values from paired *t*-test: *P < 0.05, **P < 0.01, ***P < 0.001.

**Figure S5**. **Jaccard distance between the predicted gene family profile of E. coli strains and the ground truth (Paired) is much smaller than the distance to randomly sampled reference genomes (Random)**. pWGS datasets at 1$\times$ sequencing depth with different strain numbers (2, 4, 6 and 8 strains). P values from t-test; *P < 0.05, ***P < 0.001, ****P < 0.0001.

Figure S6. Precision-Recall curve of predicted gene family profiles of *E. coli* strains. Solid line: prediction by StrainPanDA; dotted line: a randomly generated gene family profile as the control. Synthetic mixtures of *E. coli* strains: pWGS dataset, 1× sequencing depth.


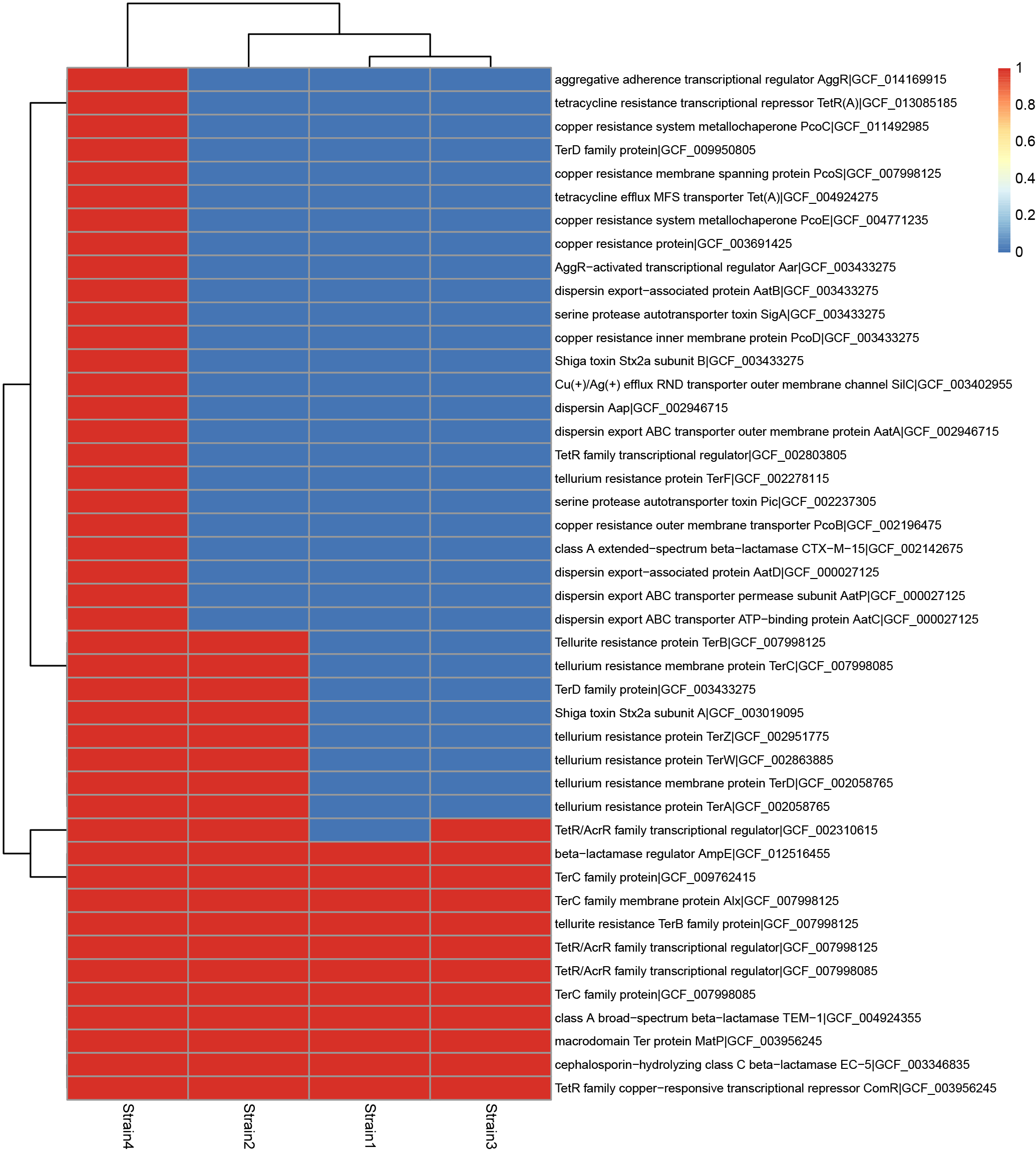


Figure S7. Identification of *E. coli* strain-specific outbreak-related gene families by StrainPanDA. Red/blue: presence/absence of a gene family. Strain4 is the *E. coli* O104 strain in synthetic mixture (Sync-O104 dataset).

**Figure S8. Benchmarking the predicted gene content profiles of StrainPanDA against PanPhlAn/PanPhlAn3. (A, C)** The heatmap of the predicted gene family profiles by StrainPanDA, PanPhlAn and PanPhlAn3 of *E. coli* strains (in pWGS dataset at 1× sequencing depth). In the heatmap, each row represents one gene family, and each column represents gene family profile of one strain. Hierarchical clustering on rows and columns are performed based on Euclidean distance. StrainPanDA: the gene content profile of the selected strain from 20 samples. PanPhlAn/PanPhlAn3: the profile from the sample with highest abundance of each selected strain. For each strain, we selected the sample where the strain had the highest abundance and use it for PanPhlAn or PanPhlAn3. Reference: the profile from PanPhlAn/PanPhlAn3 on the error-free synthetic data of the selected strain. To generate the reference gene profiles of the selected strains, especially those strains that were not included in the pangenome, we simulated error-free data of each selected strain and used the reported gene content profile from PanPhlAn or PanPhlAn3 as the reference profile. Due to the incompatible versions of COG databases PanPhlAn and PanPhlAn3 use, we compared StrainPanDA to the two tools separately. To make the results from StrainPanDA comparable with PanPhlAn3, we converted the StrainPanDA result to PanPhlAn3 annotation using blastn. Only the overlapped gene families between StrainPanDA and PanPhlAn3 were considered. **(B, D)** Jaccard distance between the predicted gene content profiles and the reference (n = 4 strains). ns: not significant, paired *t*-test.

**Figure S9. Relative abundance of B. longum in the gut microbiome of mothers and infants (3 different time points).** NB: newborn, 4M: 4-month, 12M: 12-month. P values from t-test: **P < 0.01, ****P < 0.0001.


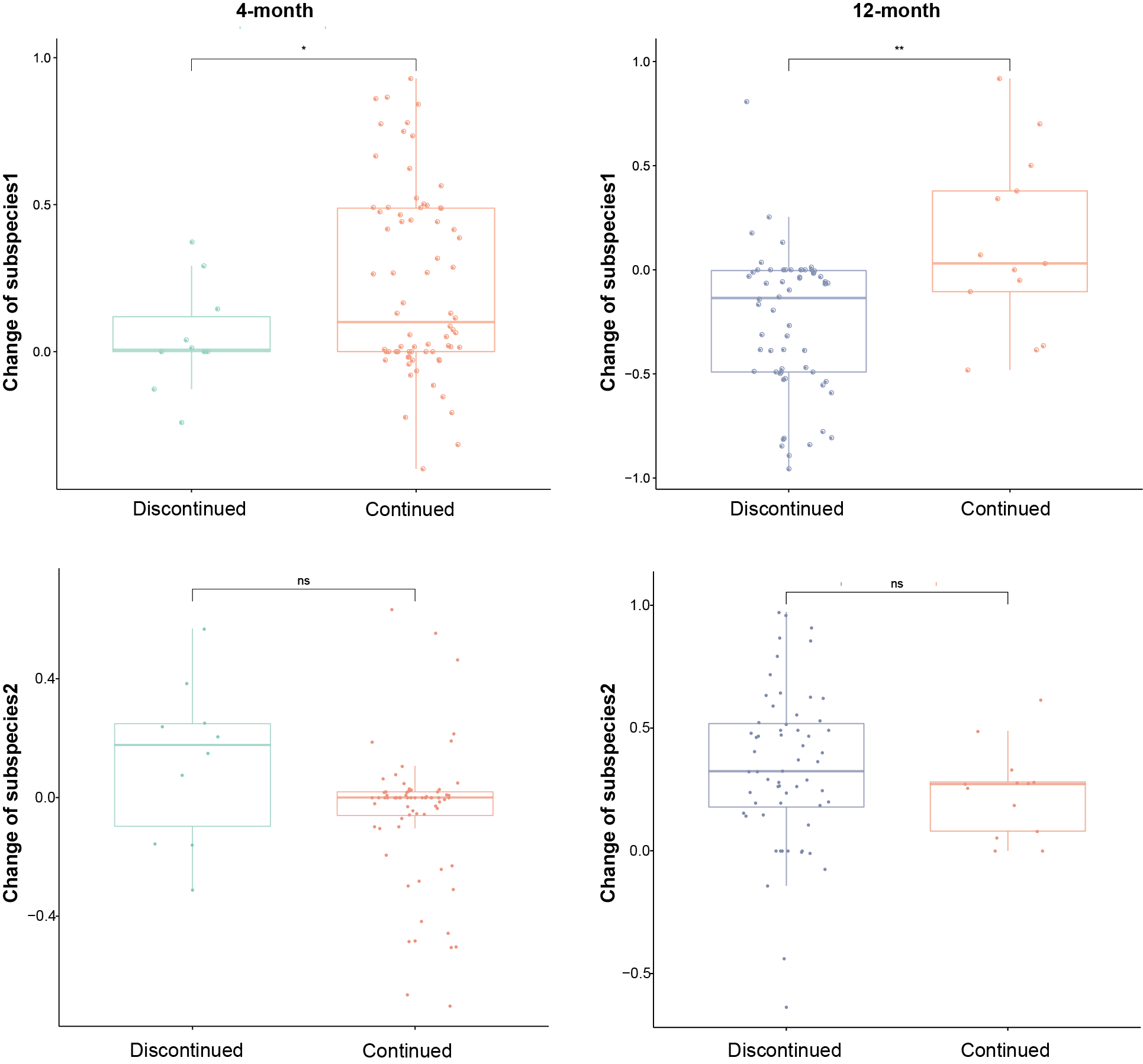


Figure S10. Difference in the relative abundance of *B. longum* subspecies 1 and subspecies 2 of infant gut microbiome between successive time points. Red: continued breastfeeding; green: discontinued at 4 months; purple: discontinued at 12 months. P values from *t*-test: *P < 0.05, **P < 0.01, ns: not significant.


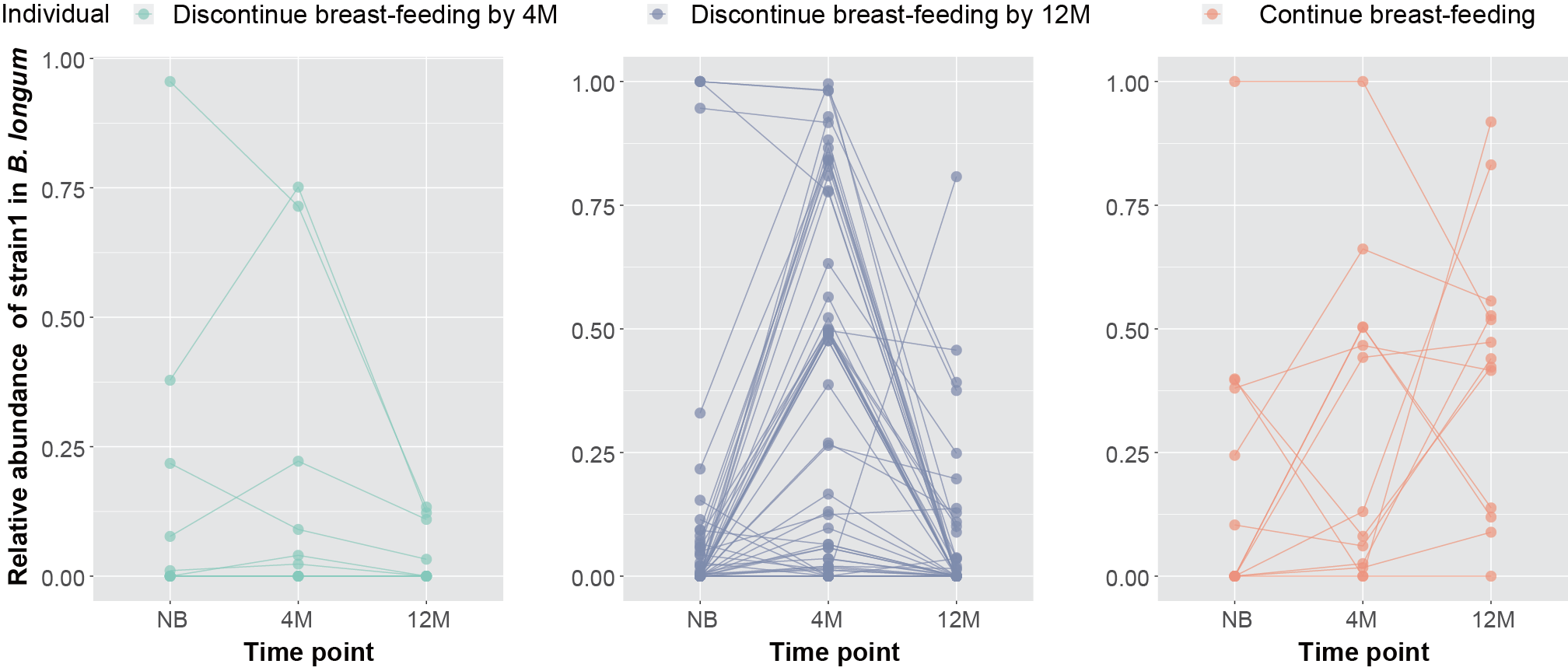


Figure S11. The dynamics of *B. longum* subspecies 1 in infant gut microbiome is affected by breastfeeding. NB: newborn, 4M: 4-month, 12M: 12-month.


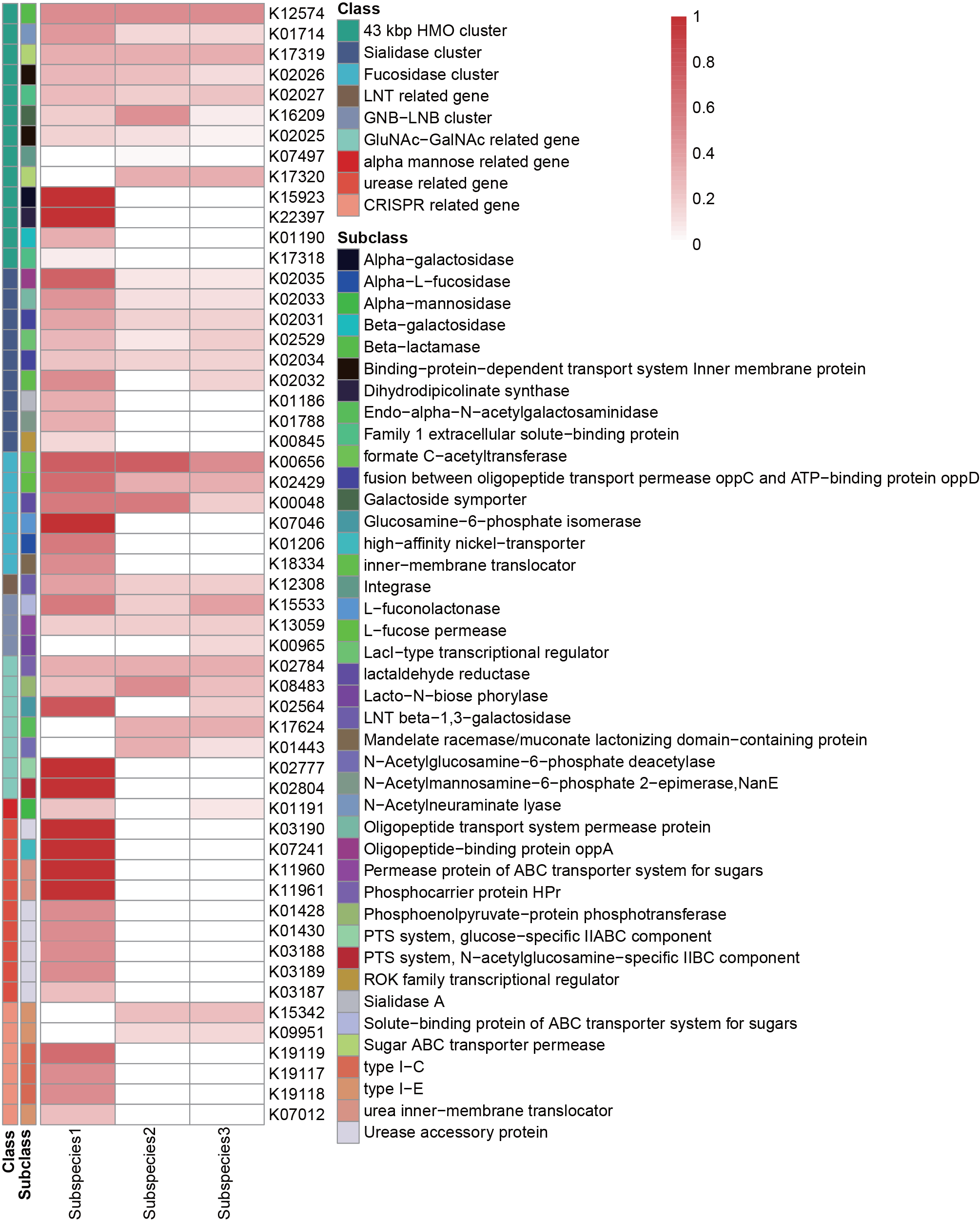


Figure S12. Functional variations of predicted *B. longum* subspecies. Gene families related to the metabolism of host glycans, urease and CRISPR are annotated by with the KEGG database. Columns are subspecies and rows are KO ID of genes. The color scale in heatmap indicates the normalized coverage of gene families of the specific KO ID.

Figure S13. Heatmap of functional variations of predicted *B. longum* subspecies on genes related to the metabolism of host glycans by CAZy annotations. Columns are subspecies and rows are CAZy families. The color scale in heatmap indicates the normalized gene coverage in the specific CAZy family.


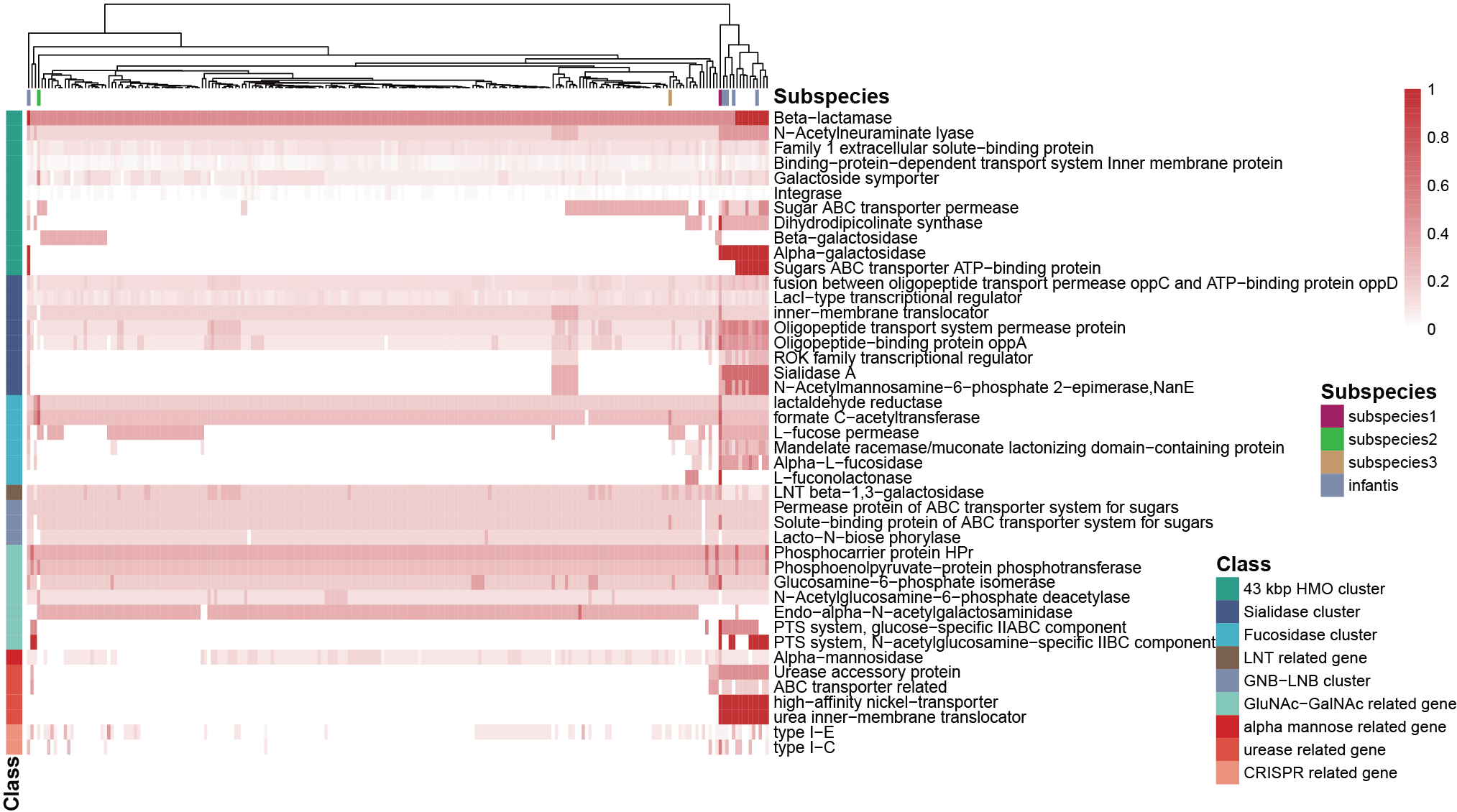


**Figure S14.** **Clustering of predicted subspecies and reference genomes of *B. longum*.** Each column was a strain/subspecies, and each row was a subclass of function. The color scale in heatmap indicates the normalized gene coverage in the specific subclass (*i.e.* the fraction of detected genes belonging to the subclass). Strains previously annotated as *B. longum* subspecies *infantis* were highlighted.

Figure S15. StrainPanDA analysis of *F. prausnitzii* in a metagenomic dataset of *Clostridium difficile* infected patients treated by fecal microbiota transplantation (FMT).


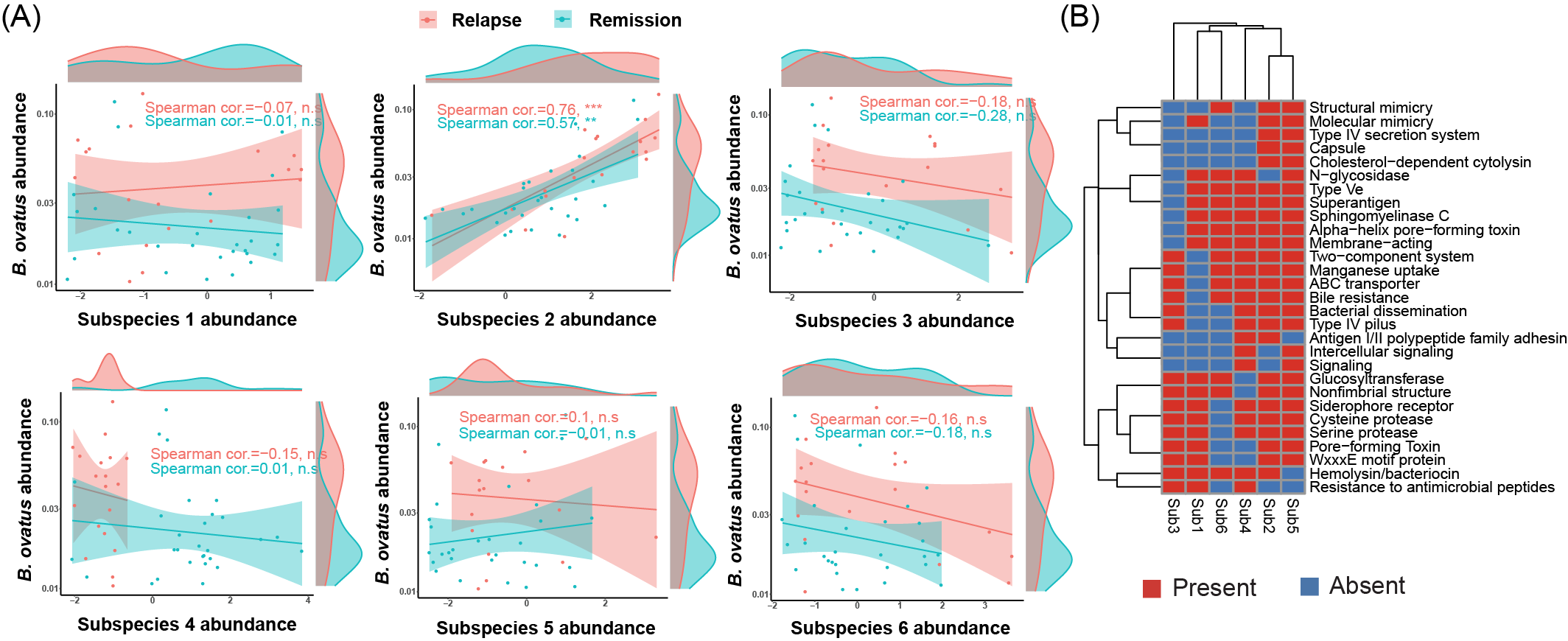


Figure S16. StrainPanDA analysis of *B. ovatus* in a metagenomic dataset of Crohn’s disease patients treated by fecal microbiota transplantation (FMT). (A) Scatter plots with fitted linear regression lines (with 95% confidence interval represented by the shaded areas) showing the relationship between the abundances of *B. ovatus* and its subspecies (normalized by centered log-ratio transformation). The density plot on the side of each panel shows the marginal distribution of the corresponding variable. (B) Virulence factor profile of the *B. ovatus* subspecies (virulence factors shared by all the subspecies were not shown).

**
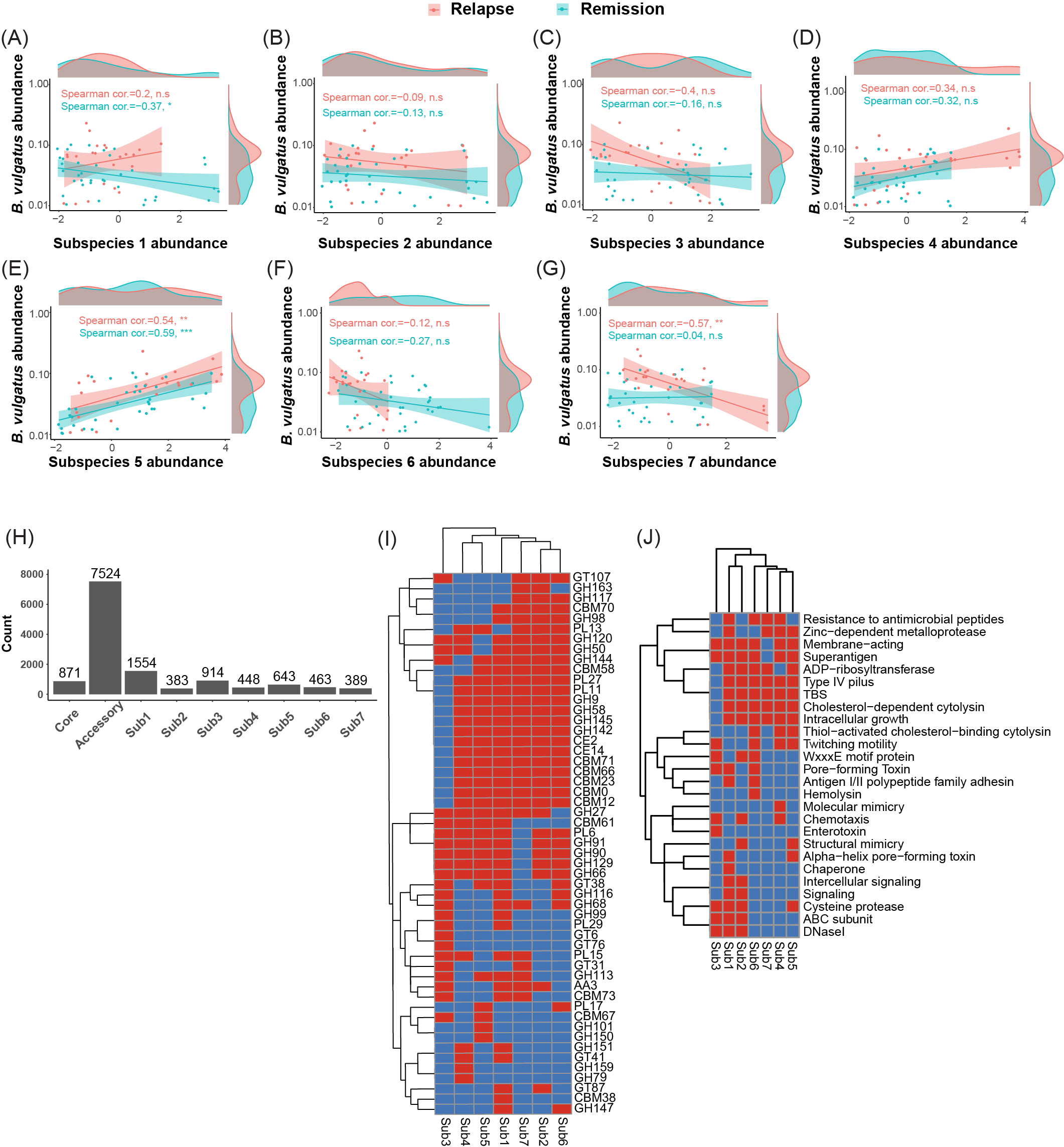
**

Figure S17. StrainPanDA analysis of *B. vulgatus* in a metagenomic dataset of Crohn’s disease patients treated by fecal microbiota transplantation (FMT). (A-G) Scatter plots with fitted linear regression lines (with 95% confidence interval represented by the shaded areas) showing the relationship between the abundances of *B. vulgatus* and its subspecies (normalized by centered log-ratio transformation). The density plot on the side of each panel shows the marginal distribution of the corresponding variable. (H) Barplot showing the summarized pangenome information of *B. vulgatus* present in the dataset. (I) Carbohydrate-Active enZYmes (CAZy) profile of the *B. vulgatus* subspecies (CAZy families shared by all the strains were omitted). (J) Virulence factor profile of the *B. vulgatus* subspecies (virulence factors shared by all the subspecies were not shown).

**Figure S18. Evaluation of StrainPanDA using synthetic data with varying sample size.** JSD quantifies the accuracy of predicted strain composition, while AUPRC quantifies the accuracy of predicted gene content profile of strains. P values from t-test: *P < 0.05, ***P < 0.001, ****P < 0.0001. JSD: Jensen-Shannon divergence; AUPRC: area under the precision-recall curve.

**Figure S19. Runtime analysis of StrainPanDA.** To evaluate the time complexity of StrainPanDA, we ran StrainPanDA on synthetic *E. coli* datasets of different strain numbers and sample sizes using a desktop with 8 threads and 16GB of RAM (**Methods**). **(A)** Runtime (seconds) as a function of number of samples. The strain number was fixed at 4. For each sample number, 3 replicates were used. **(B)** Runtime (seconds) as a function of strain number, with sample number fixed at 20 in each dataset. For each strain number, 1 sample with no background (from pWGS at 1× sequencing depth) and 3 samples with 25-fold background (from IBD, FMT and MI separately) were used. The results showed that the running time increased by three times (from ~100 seconds to 300 seconds) when the sample number increased by eight times (from 5 to 40), and increased by 2.5 times (from ~200 to 450 seconds) when the strain number increased from 2 to 8.
